# Ophthalmic Metastasis of Breast Cancer and Ocular Side Effects from Breast Cancer Treatment and Management: Mini Review

**DOI:** 10.1155/2015/574086

**Published:** 2015-05-11

**Authors:** Ilias Georgalas, Theodore Paraskevopoulos, Chryssanthi Koutsandrea, Evgenia Kardara, Panagiotis Malamos, Dimitrios Ladas, Dimitris Papaconstantinou

**Affiliations:** Department of Ophthalmology, “G. Gennimatas” Hospital of Athens, University of Athens, 154 Mesogeion Avenue, 11527 Athens, Greece

## Abstract

Breast cancer is one of the most common malignant diseases occurring in women, and its incidence increases over the years. It is the main site of origin in ocular metastatic disease in women, and, due to its hematogenous nature of metastatic spread, it affects mainly the uveal tissue. The purpose of this paper is to summarize the clinical manifestations of the breast cancer ocular metastatic disease, alongside the side effects of the available treatment options for the management and regression of the systematic and ophthalmic disease.

## 1. Introduction and Epidemiology

Breast cancer is the most common neoplastic disorder diagnosed in women [1]. It is classified as the second most frequent cause of death in women, after lung cancer [1]. Despite recent advances in early diagnosis and effective treatment, it is estimated that up to one-third of patients having been diagnosed with breast cancer will develop metastatic disease [2]. In contrast, breast cancer in men, male breast cancer (MBC), is rare and accounts for less than 1% of total neoplastic cases in male population and about 1% of all breast cancer diagnoses [3, 4]. Male breast cancer's incidence is increasing over the years, due to lack of awareness of male population regarding this disease. As a result, male patients are diagnosed in a more advanced stage of the disease [5]. Ocular metastases of breast cancer, although rare, can occur in both male and female patients, and in women breast is the most common site of origin of ocular metastatic tumors, since, in 49% of patients with ocular metastatic disease, the primary tumor origin was the breast [6].

*Breast Cancer Ocular Metastatic Disease*. Metastatic tumor accounts for the most common ocular malignancy [7]. The uveal tract is considered to be one of the most favored sites where breast cancer metastases develop [8]. The incidence of ocular breast cancer metastatic disease presents variations among different studies, with rates between 5 and 30% [9, 10], attributed to the asymptomatic nature of ocular metastatic foci, in contrast to metastatic disease in other organs [11]. Metastatic foci of the disease in the lungs, central nervous system, or bones are usually detected prior to diagnosis of ocular metastases [12, 13]. In fact, the only significant risk factor for the development of ocular malignant foci in patients with breast cancer is the dissemination of the disease in lungs and brain [11]. In rare cases ocular metastasis can represent the initial manifestation of an undiagnosed breast tumor [14]. The survival rate of patients with ophthalmic metastatic disease depends on the level of organ dysfunction caused by dissemination of the tumor [15].

## 2. Clinical Signs and Manifestations

Since the vast majority of ocular metastatic sites are created by hematogenous spread [16], the uveal tissue (iris, ciliary body, and choroid), especially the choroid, is the primary ocular site of breast cancer metastases. Involvement of the uvea occurs in up to 10% of cases with metastatic breast cancer: the highest metastatic efficiency index in comparison to all body tissues [17]. The vascularity of the choroid and microenvironmental factors have been proposed as possible explanations for increased metastatic dissemination in the choroid [18]. The choroid is predominantly affected with an incidence of 81% of ocular metastasis, followed by the iris with 9%, optic disc with 5%, and ciliary body with 2% [19]. The most common clinical complaint of patients with ocular metastatic disease is blurred vision, ocular pain, visual field defects, metamorphopsia, floaters, and photopsia [6]. In patients with diagnosed ocular metastatic disease, the risk of fellow eye involvement is estimated to be 5% in a period of ten months after diagnosis [20].

### 2.1. Symptoms and Signs of Metastatic Choroidal Disease

**Figure 1:**
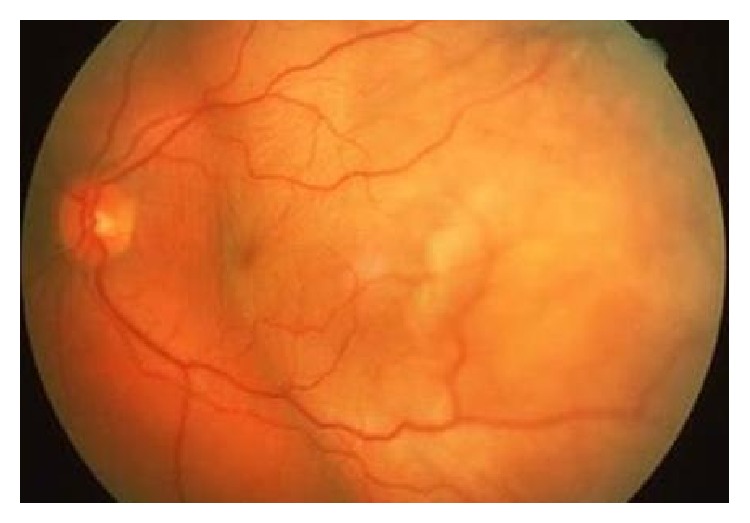
Fundus photo of the left eye of a patient demonstrating metastatic tumor involving the macula.

**Figure 2:**
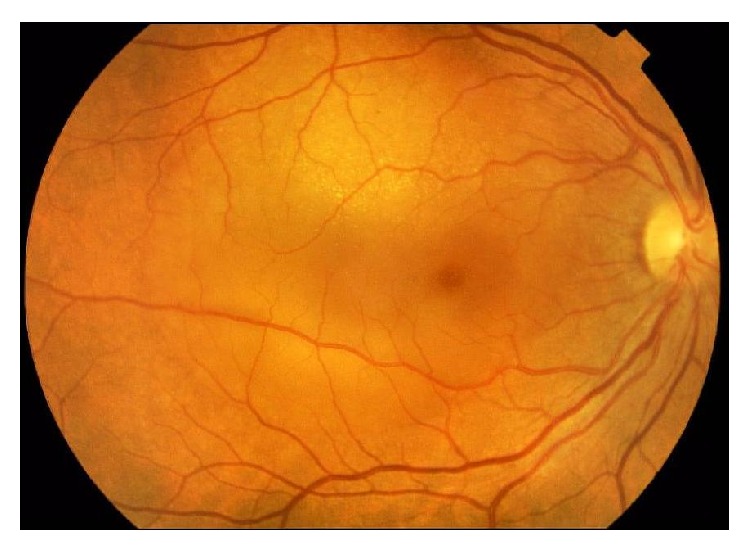
Fundus photo of the right eye of a patient with metastatic disease. Please note the tumor close to the macular area and the adjacent shallow retinal detachment involving the macula.

Most patients with metastatic choroidal disease (81% of uveal metastases) are asymptomatic, and the most common symptom they develop is visual impairment with or without metamorphopsia [21]. The visual deterioration is caused by macular involvement of the malignant lesions (Figure 1), the presence of subretinal fluid in the fovea, or the induced hyperopia (Figure 1) [22]. Choroidal foci of metastases are typically located between the macula and the equator and are presented in fundoscopy as homogenous, creamy-yellow, plateau shaped lesions which spread laterally across the choroid [20]. In addition, they are usually complicated with serous foveal detachments and retinal pigment epithelium (RPE) alterations, appearing fundoscopically as deposits on the surface of the tumor with golden-brownish appearance (Figure 2) [23]. As in most metastatic diseases, tumors arising from breast cancer metastases are multifocal and are mainly located in the superotemporal quadrants of the fundus [22]. Choroidal metastases may also lead to an exudative retinal detachment, characterized by shifting patterns of subretinal fluid depending on the posturing of the patient (Figure 2) [19, 24].

### 2.2. Symptoms and Signs of Iris Metastasis

Clinical presentation of iris metastases (9% of uveal metastases) includes ocular pain rather than visual impairment, if associated with secondary glaucoma or iridocyclitis [25]. The decreased visual acuity is secondary to anterior chamber seeding or to cataract formation. On slit lamp examination, they are identified as rapidly growing yellow to white solitary iris nodules with pupillary distortion, located in the iris (most commonly the midperiphery of the inferior quadrant) and in some cases infiltrating the trabecular meshwork [26, 27]. More rarely they may be accompanied by pseudohypopyon [27].

### 2.3. Symptoms and Signs of Optic Disc Metastasis

Optic disc involvement is due either to direct extension of a choroidal tumor which is located close to the optic disc or to hematogenous spread of neoplastic cells to the circulation of the papilla [28]. Ophthalmoscopically, it appears as a diffuse yellow-white thickening of the optic nerve head in 84% of cases and as a distinct nodular lesion in 16%, with minimal extension to the nerve fiber layer and secondary disc edema [21, 28].

### 2.4. Symptoms and Signs of Metastatic Ciliary Body Disease

Ciliary body metastases (2% of uveal metastases) are most commonly located in the inferior quadrant of the eye (25% of cases) and form cyst shaped or sessile masses, which are very difficult to be identified directly [29]. The ciliary body may be affected either directly or by an extension of a choroidal or iris tumor. Major complications of ciliary body masses include shallowing of the anterior chamber, cataract, and lens subluxation [24]. Common signs of ciliary body involvement are conjunctival and/or episcleral hyperemia, iridocyclitis, glaucoma, pseudohypopyon, and hyphaema [24].

### 2.5. Extraocular Metastasis

#### 2.5.1. Orbital

The extraocular muscles represent the main site of breast cancer orbital metastasis, causing pain, proptosis, and diplopia. They are identified pathologically as solid deposits of the muscles [30]. Orbital metastasis may cause exophthalmos, from mass effect, or enophthalmos, when infiltrated muscles lead to posterior pulling of the eye [31, 32]. Sporadic cases of breast cancer migration to the conjunctiva [30], the eyelids [33], and the cranial nerves [34] have been reported.

#### 2.5.2. Cerebral

Brain is a common location for metastasis secondary to breast cancer, usually affecting the cortical and juxtacortical zones [35]. Clinical symptoms of brain insult include seizures, headache, and focal motor or mental syndromes [35]. As far as vision is concerned, patients may experience symptoms such as visual field defects like homonymous hemianopsia, and in certain cases dyschromatopsia [36, 37].

### 2.6. Paraneoplastic Manifestations

Paraneoplastic manifestations of breast cancer in the eye are not common. They are considered as immunological responses against the antigens of the tumor—expressed by normal cells—rather than metastatic processes [38]. They may cause diplopia, nystagmus, and loss of vision [39, 40].

## 3. Diagnosis

**Figure 3:**
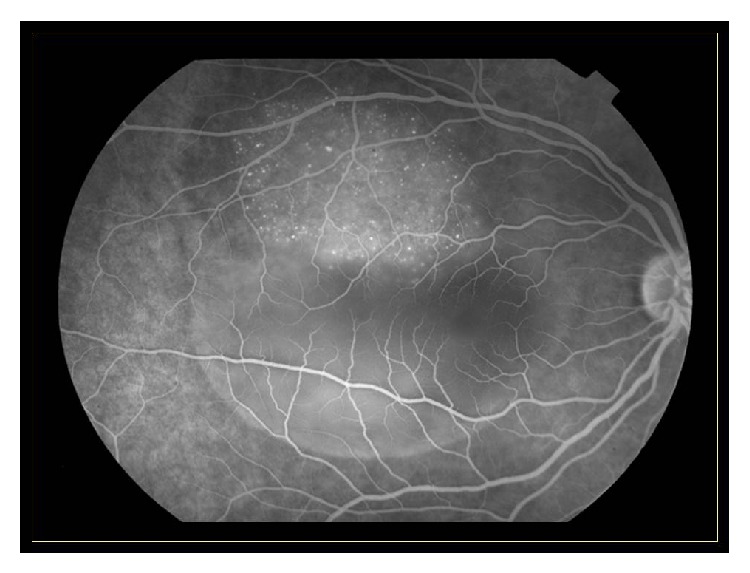
Fluorescein angiography of the same patient presented in Figure 2.

**Figure 4:**
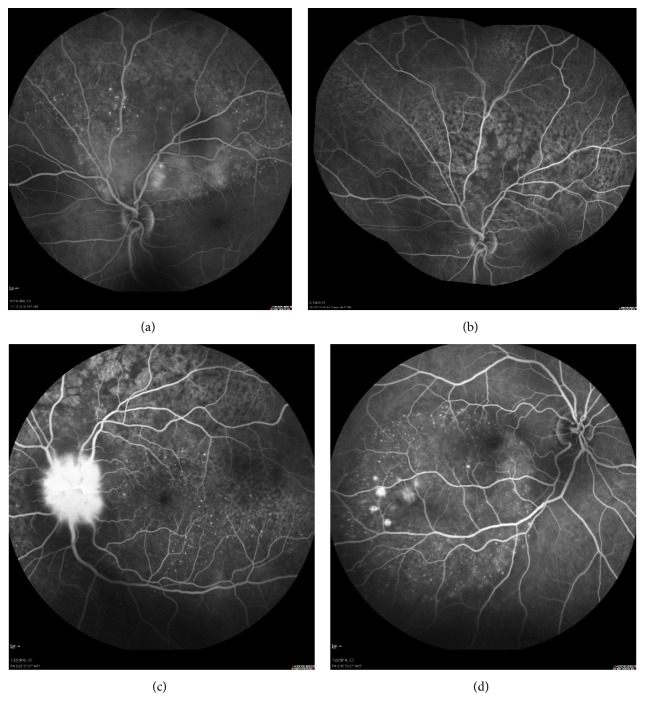
Angiographic imaging of a woman with metastatic breast cancer and choroidal disease. (a) Choroidal metastases presenting as pinpoint hyperfluorescence and dye pooling under the area of neurosensory detachment. (b) Resolution of angiographic signs after systemic chemotherapy. Retinal pigment epithelium alteration resembling “leopard skin” pattern. (c) Relapse of metastatic disease with infiltration of the optic disc. Diffuse late leakage in the peripapillary area. (d) Involvement of the fellow eye with small PEDs presenting pooling and numerous pinpoints.

**Figure 5:**
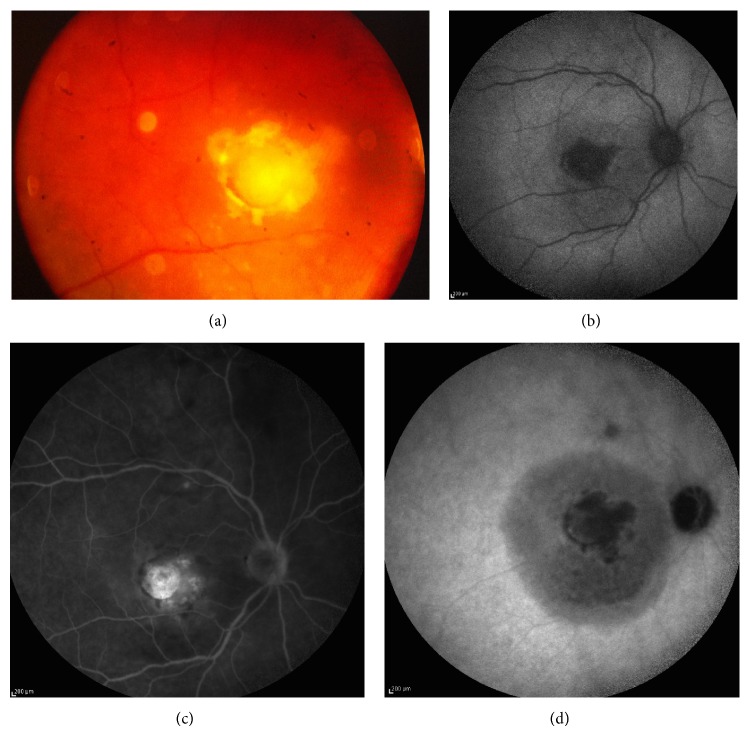
Infectious infiltration of the macula due to contamination with* Staphylococcus epidermidis* of venous fistula in a 45-year-old woman with breast cancer. (a) Creamy-white circular infiltration of the macula in a 45-year-old woman with breast cancer. (b) Fundus autofluorescence image with central defect. (c) Fluorescein angiography with staining of the central defect. (d) ICGA in late phase reveals a wider ring of retinal pigment epithelium involvement surrounding the central lesion.

Diagnosis of ocular metastatic disease is mainly clinical. Slit lamp biomicroscopy is the hallmark for identifying metastatic sites, alongside history of breast cancer. Additional imaging studies are indicated in cases of doubt. More specifically, fluorescein angiography, fundus autofluorescence (FAF), and optical coherence tomography (OCT) are useful diagnostic tools for diagnosis of choroidal metastases (Figures 3–5) [41]. The fluorescein angiography reveals hyperfluorescence of the mass in the late venous phase [41]. Fundus autofluorescence provides tumor images as hyperfluorescent areas of focal pigmentation and subretinal fluid, with hypofluorescent margins [41]. Fourier-domain OCT demonstrates elevation of the retinal pigment epithelium (RPE) and retina, retinal thickening, and areas of retinal detachment, if present [41]. Additionally, B-scan ultrasonography demonstrates metastatic masses as areas with medium to high internal reflectivity [21]. The magnetic resonance imaging (MRI) contributes to differential diagnosis of breast cancer metastatic disease and choroidal melanoma, since choroidal melanoma demonstrates high signal intensity on T1-weighted images [42]. More recently, positron emission tomography-computed tomography scan (PET-CT scan) has been reported to contribute to identification of breast cancer choroidal metastatic disease [43].

Before deciding the exact treatment modalities that will be applied to each patient, it is essential to identify the primary source of the metastatic disease [44]. Although in most cases diagnosis of breast cancer is established at the time of ocular metastasis [12, 13], cases of ocular metastasis of unknown origin require histopathological confirmation with the performance of ocular tumor biopsy [45]. Moreover, intraocular biopsy may provide additional information about the nature of the tumor itself, contributing to the optimal therapeutic strategy. Specifically, intraocular biopsy may identify Her2/neu-positive patients that may benefit from adjuvant anti-Her2/neu therapy [46] and estrogen or progesterone receptor-positive tumor cells that would respond to endocrine therapy administration [47].

## 4. Ocular Side Effects of Systemic Breast Cancer Treatment

Therapeutic management of breast cancer involves systemic treatment [48] and/or local therapy [49]. Systemic therapy may lead to ocular tumor control as well, but in certain cases additional local treatment is required [48]. Systemic treatment includes hormone therapy, specifically selective estrogen receptor modulator (tamoxifen), aromatase inhibitors (anastrozole, letrozole, and exemestane), cytotoxic chemotherapy, or targeted therapy with monoclonal antibodies (trastuzumab) [50]. The aim of local treatment is to preserve patients' vision and improve their quality of life.

The treatment plan against ocular metastatic foci requires collaboration of ophthalmologists with oncologists and neuroradiologists, in order to define the optimal therapeutic approach for each patient, with the less possible side effects. Indications for ocular metastases treatment include visual deterioration due to metastatic tumors, location of metastases close to the macula or the optic nerve, enlargement of the neoplastic lesions despite systemic therapy, and lesions causing intolerant pain to patients [21].

### 4.1. Endocrine Treatment

The majority (60–70%) of breast cancers in postmenopausal women express estrogen or progesterone receptors; thus they are susceptible to endocrine therapy [51]. The endocrine therapy consists of two regimens: selective estrogen receptor modulators (tamoxifen) and aromatase inhibitors.

#### 4.1.1. Tamoxifen

Tamoxifen is a competitive antagonist of estrogen at its receptor site [52]; for premenopausal women with metastatic disease, tamoxifen is considered as the treatment of choice. Lower doses of tamoxifen (20 mg/day) are currently used in order to avoid complications that have been reported in the past such as retinal toxicity (tamoxifen retinopathy: white, refractile deposits in nerve fiber and inner plexiform layers of the retina), optic neuritis, and corneal disease (tamoxifen keratopathy: “whorl-like” deposits on the cornea) and had led to discontinuation of the drug [52, 53].

#### 4.1.2. Aromatase Inhibitors

In postmenopausal women, estrogens originate from adrenal androgen's peripheral conversion and aromatase inhibitors interfere in this path by preventing the conversion [52]. The third generation aromatase inhibitors (anastrozole, letrozole, and exemestane) have proven clinical efficacy in metastatic breast carcinoma [54]. Systemic side effects have been reported less commonly in comparison to tamoxifen and ocular toxicity has not been associated with the administration of these drugs [55].

### 4.2. Cytotoxic Treatment

The most commonly administered regimens are CMF (cyclophosphamide, methotrexate, and 5-fluorouracil), FEC (5-fluorouracil, epirubicin, and cyclophosphamide), and AC (doxorubicin and cyclophosphamide) with or without the incorporation of taxanes such as docetaxel or paclitaxel [56]. Usual side effects of cytotoxic agents include blepharospasm [57], blurred vision [58], excessive lacrimation [59], and keratitis [60]. The symptoms usually appear up to 14 days after initiation of chemotherapy and do not last after the discontinuation of treatment [61].

### 4.3. Monoclonal Antibodies

Conventional chemotherapeutic modalities are targeted not only against neoplasmatic cells, but against normal cells as well, causing numerous side effects. This fact led to development of targeted therapies, which aim at molecular pathways involved in tumor cells proliferation [62]. The human epidermal growth factor receptor (HER2) is overexpressed in 25% of breast cancer cases and is associated with more aggressive phenotype [62]. A humanized monoclonal antibody (trastuzumab), which binds to the extracellular juxtamembrane of this receptor, blocks the activation of HER2. It has been reported that combined with other systemic therapies it has led to regression of ocular metastatic disease [63, 64]. Ocular side effects of trastuzumab have not been reported.

## 5. Local Management of Metastatic Eye Disease

Local treatment's aim is to preserve patients' vision and improve their quality of life. It is administered in addition to systemic treatment regimens. Local treatment modalities include radiotherapy, laser application, intravitreal antivascular endothelial growth factors (anti-VEGF), photodynamic therapy, and enucleation of the eye.

### 5.1. Radiotherapy

External beam radiation therapy (EBRT) is the first and most widely applied local treatment modality against ocular metastatic disease [65]. It contributes to size regression of both choroidal and iris tumors, by damaging the DNA of the neoplasmatic cells [66]. Usual dosage applied varies between 20 and 50 grays. Regression of the metastatic tumor has been reported in 63–83% of cases [21]. EBRT is a treatment method which induces several ocular side effects. The most common ophthalmic complications are skin erythema, conjunctivitis, cataract formation, exposure keratopathy, iris neovascularization, and radiation retinopathy and papillopathy [67]. Patients' survival for over six months after EBRT application is the most important factor for developing side effects [68].

### 5.2. Alternative Radiation Treatment

Proton beam radiotherapy has been tested for localized intraocular irradiation [69]. Due to the nature of charged particles used, highly localized dose distribution is possible. Since protons deposit their energy at the end of their range, a lower number of radiation treatment fractions are needed, in comparison to EBRT. In addition, lower volume of irradiation is delivered to the surrounding tissues, resulting in less ocular toxicity than EBRT [70]. The results of the method were promising (tumor regression in 84% of cases) for improving patients' quality of life [69]. Side effects were reported in a lower rate than EBRT (29%), most commonly cataract, keratitis, and radiation maculopathy/papillopathy [69].

### 5.3. Chemotherapy and Hormone Therapy

The application of systemic chemotherapy and hormonal therapy in patients with systematic, extraocular metastasis has demonstrated satisfactory results in managing metastatic eye disease, leading even to complete regression of choroidal metastasis [70]. Its efficacy, though, is only demonstrated in individual case reports and not through extensive trials.

### 5.4. Laser Treatment

Several types of lasers have been applied, with beneficial outcome against uveal metastatic disease [71]. Initially, transpupillary thermotherapy (TTT), in which a modified diode laser is used to deliver heat to choroid and RPE through the pupil, results in tumor necrosis [72]. In addition, laser photocoagulation with the use of argon or krypton causes occlusion of the vessels of the tumor which is followed by tumor necrosis [72]. It appears to be effective with no ocular complications, but it is applied in small sized metastatic foci [71].

### 5.5. Antivascular Endothelial Growth Factor-Targeted Treatment

Bevacizumab (Avastin) is a monoclonal antibody that blocks all VEGF-A isoforms. It was the first anti-VEGF therapy approved by the FDA for the treatment of colorectal, breast, and lung cancer [73]. Recent case series studies have shown promising results, since they demonstrated that bevacizumab is effective as a treatment option in patients with metastatic choroidal disease, unresponsive to systemic therapy. Its role is in preventing visual loss by achieving choroidal tumor regression [74]. Its efficacy is based on the antiangiogenic and antipermeability properties of bevacizumab [74]. More extensive trials are required for the establishment of the effectiveness and the determination of the exact treatment protocol.

### 5.6. Photodynamic Therapy

Photodynamic therapy (PDT) has lately been applied for the local regression of choroidal metastatic disease with promising outcomes, since its complete tumor control is reported in 78% of cases [75]. It achieves tumor destruction by producing highly reactive oxygen species and by activating immune response against tumor cells. In addition, the substance administered during PDT performance is verteporfin, which binds to vascular endothelial cells and leads to intraluminar thrombosis. The increased vascularity that characterizes the choroidal metastases explains the initial efficacy of the method [75].

### 5.7. Enucleation

Enucleation is only used as a treatment option for eyes with intolerable pain from secondary glaucoma development [23].

## 6. Prognosis

Despite recent advances in diagnosis and treatment modalities, the prognosis of breast cancer metastatic disease remains poor with estimated median life expectancy of 6 to 9 months [76].

## 7. Conclusions

Breast cancer is the most common malignancy in women. The incidence of breast cancer is increasing especially in developed world. Better screening, earlier detection, and better treatment modalities have positively altered the prognosis and survival time of patients suffering from breast cancer. This subsequently leads to an increased variety of ocular manifestations and problems associated with patients' vision that are likely to require ophthalmological consultation and management. Because early diagnosis and prompt management may positively alter the prognosis for these patients, ophthalmologists should be aware of this entity.

To date, the treatment of choice for ocular metastasis is radiotherapy. Chemotherapeutics also play an important role in the control of uveal metastasis. The use of anti-VEGF therapy is almost routine in the ophthalmology practice for many ocular diseases like age related macular degeneration, diabetic retinopathy. Its role in the treatment of uveal metastasis remains to be determined as more reports become available. However it may help avoid the need for other more damaging treatments.
